# Collagen Scaffolds Containing Hydroxyapatite-CaO Fiber Fragments for Bone Tissue Engineering

**DOI:** 10.3390/polym12051174

**Published:** 2020-05-20

**Authors:** Shiao-Wen Tsai, Sheng-Siang Huang, Wen-Xin Yu, Yu-Wei Hsu, Fu-Yin Hsu

**Affiliations:** 1 Graduate Institute of Biomedical Engineering, Chang Gung University, Taoyuan City 333323, Taiwan; swtsai@mail.cgu.edu.tw; 2 Department of Periodontics, Chang Gung Memorial Hospital, Taipei 105406, Taiwan; 3 Department of Bioscience and Biotechnology, National Taiwan Ocean University; Keelung City 202301, Taiwan; mm0070360@gmail.com (S.-S.H.); andy54861@yahoo.com.tw (W.-X.Y.); qazwest74@gmail.com (Y.-W.H.)

**Keywords:** collagen, hydroxyapatite, fiber fragments, composite, scaffold, bone regeneration

## Abstract

Collagen (COL) and hydroxyapatite (HAp) are the major components of bone, therefore, COL-HAp composites have been widely used as bone substitutes to promote bone regeneration. We have reported that HAp-CaO fibers (HANFs), which were fabricated by a sol-gel route followed by an electrospinning technique, possessed good drug-loading efficiency and limited the burst release of tetracycline. In the present study, we used HANF fragments to evaluate the effects of COL-HANF scaffolds on MG63 osteoblast-like cell behaviors. COL-HANF composite scaffolds in which the average diameter of HANFs was approximately 461 ± 186 nm were fabricated by a freeze-drying process. The alkaline phosphatase activity and the protein expression levels of OCN and BSP showed that compared with COL alone, the COL-HANF scaffold promoted the differentiation of MG63 osteoblast-like cells. In addition, the bone regeneration ability of the COL-HANF scaffold was examined by using a rabbit condylar defect model in vivo. The COL-HANF scaffold was biodegradable and promoted bone regeneration eight weeks after the operation. Hence, we concluded that the COL-HANF scaffold has potential as a bone graft for bone tissue engineering.

## 1. Introduction

Tissue engineering interconnects many disciplines, such as materials science, bioengineering, molecular and cell biology, and clinical medicine, to develop functional alternatives for impaired tissues [1]. Cells, bioactive molecules and scaffolds are the three major components used in tissue engineering [2]. Three-dimensional scaffolds are prepared to support cell attachment, proliferation and differentiation for bone tissue engineering [3].

Bone graft substitutes, such as autologous bone grafts, allogeneic bone grafts and synthetic bone grafts, have been used for the treatment of large bone defects caused by congenital diseases, injuries, or trauma [4]. Autologous bone grafts are the preferred clinical option but suffer from limited tissue availability and donor-site morbidity. Allogeneic bone grafts are obtained from cadavers in tissue banks and thus pose a risk of disease transmission or undesired immunological rejection [5]. Therefore, it is important to develop suitable synthetic bone grafts to overcome the drawbacks of autologous and allogeneic bone grafts [6,7].

An ideal synthetic bone graft must be biocompatible and biodegradable and support osteoconduction, osteoinduction and osteointegration [8]. A mature bone matrix is composed of minerals, predominantly hydroxyapatite (HAp), and proteins such as type I collagen (COL). COL scaffolds have been widely used as bone grafts because they can partially mimic the extracellular matrix (ECM) of bone [9]. However, the main drawback of COL scaffolds is their poor mechanical properties [10]. Therefore, various methods have been developed to improve the mechanical properties of the COL matrix [11,12,13].

The incorporation of bioceramics, such as bioglass nanoparticles [14], bioglass nanofibers [15] and HAp nanoparticles [16], is a popular method to reinforce COL scaffolds. Bioceramics have not been investigated as a means to improve mechanical properties, but they are bioactive, which accelerates mineralization. Venugopal et al. noted that compared with COL scaffolds, HAp-COL scaffolds enhanced the attachment and proliferation of osteoblast cells [17]. Kim noted that bioglass nanofiber-COL composites exhibited excellent bioactivity in vitro and promoted alkaline phosphatase (ALP) activity in osteoblastic cells [18]. Hsu also found that a mesoporous bioactive glass nanofiber-COL scaffold provided a suitable environment for attachment to the cytoskeleton and promoted the differentiation and mineralization of MG63 osteoblast-like cells [19].

In our past research, hydroxyapatite-CaO fibers (HANFs) fabricated by a sol-gel/electrospinning technique were demonstrated to have improved drug-loading efficiency and to retard the burst release of tetracycline and maintain antibacterial activity for a period of seven days [20]. However, the effect of HANFs on the osteogenic differentiation of osteoblast cells has not been evaluated. Therefore, we incorporated HANF fragments into COL to fabricate a collagen-HANF (COL-HANF) composite scaffold. The physical properties of the HANF fragments, such as their microstructure, average pore diameter, and phase composition, were analyzed by scanning electron microscopy (SEM), transmission electron microscopy (TEM), X-ray diffraction (XRD) and nitrogen adsorption-desorption isotherm analysis. The mechanical properties of the COL-HANF scaffold were tested by a universal mechanical tester. The cellular behavior of osteoblast-like cells on the COL-HANF scaffold was assessed using MTT assays, alkaline phosphatase activity analysis and osteoblast-specific marker protein immunofluorescence staining. In addition, the capacity of the COL-HANF scaffold to support in vivo bone regeneration and reconstruction was evaluated in a condylar bone defect model.

## 2. Materials and Methods

### 2.1. Synthesis and Characterization of HANFs

The HANFs used in the present work were synthesized based on our previously reported method [20]. First, 1.0 g of poly(ethylene glycol)-poly(propylene glycol)-poly(ethylene glycol) (P123, MW = 5800, Sigma-Aldrich Chemical Company, St. Louis, MO, USA) and 6.172 mL of triethyl phosphite (TEP, Merck, Darmstadt, Germany) were dissolved in 10 mL of aqueous ethanol solution (50% v/v) until a clear solution was obtained. A precursor solution was prepared by slowly adding calcium nitrate (Sigma-Aldrich) solution (14% in absolute ethanol) dropwise to the above TEP/P123 solution with stirring. Subsequently, the precursor solution was tightly capped and placed in an oven at 60 °C for 12 h. Next, 1.5 g of poly(vinyl pyrrolidone) (PVP, MW = 40,000, Sigma-Aldrich) and 0.45 g of P123 were dissolved in 7 mL of absolute ethanol and then added to 3 mL of the precursor solution to obtain a transparent mixture. An electrospinning system was used to fabricate a nonwoven fiber substrate. The above transparent mixture was placed in a syringe fitted with a needle (18 G) to supply a steady flow rate (1.2 mL/h). An electrical field (1.3 kV/cm) was applied between the needle and a grounded collector using a high-voltage power supply (SL 60^®^, Spellman, New York, NY, USA). The needle-to-collector distance was approximately 20 cm. The nonwoven fiber structures were collected on a grounded collector and then calcined at 800 °C for 1 h with a heating rate of 2.5 °C per min under a nitrogen atmosphere, followed by air cooling under the ambient atmosphere to obtain the HANFs.

### 2.2. Characterization of the HANFs

The morphology and average diameter of the HANFs were observed and analyzed using SEM. Briefly, the HANFs were sputter-coated with gold and observed by SEM at an accelerating voltage of 15 kV. The SEM images were analyzed with ImageJ (NIH, Bethesda, MD, USA), to determine the average fiber diameter. The average fiber diameter and standard deviation were calculated from 50 random measurements. The crystalline phase of the HANFs was characterized by XRD (Bruker D2-Phaser, Madison, WI, USA), with Cu-Kα1 radiation (λ = 1.5406 Å). All data were recorded over an angular range (2θ) of 20°~60° with a step size of 0.04° and a speed of 5°/min. The nitrogen gas adsorption-desorption isotherm was performed using a Micromeritics ASAP 2020 surface area analyzer (Micromeritics, Norcross, GA, USA) at −196 °C for surface area and pore size analyses. Before the measurement, sample was outgassed at 350 °C for 12  h in a vacuum. Surface area was calculated according to Brunauer–Emmett–Teller (BET) method and the pore size distribution was calculated according to Barrett–Joyner–Halenda (BJH) algorithm [21].

### 2.3. Fabrication and Characterization of the COL-HANF Scaffold

The COL, which was prepared from calf skin as previously described [22] was solubilized in 0.05 M acetic acid, and the pH was adjusted to 7.0 using a 1.5 M Na_2_HPO_4_ solution. The nonwoven HANF structures were reduced to fragments using a sonicator (Qsonica, Q700 sonicator, Newtown, CT, USA) for 1 min (total output energy: 51 J). The HANF fragments were mixed and suspended in a neutral COL solution with a weight ratio of 35:65 (COL to HANFs) at 4 °C. The COL or COL-HANF scaffolds were prepared following gelation and lyophilization [19]. Briefly, 0.5 mL of the COL or HANF/COL solution was added to the wells of a plate (1.9 cm^2^) and incubated at 37 °C for 6 h to form a gel. Subsequently, the COL or HANF/COL gel was freeze-dried to obtain a porous COL or COL-HANF scaffold. The COL and COL-HANF scaffolds were cross-linked using 50 mM N-hydroxysuccinimide and 50 mM 1-ethyl-3-(3-dimethylaminopropyl)-carbodiimide in absolute ethanol for 12 h. The cross-linked scaffold was washed repeatedly with absolute ethanol and then lyophilized to obtain the COL and COL-HANF scaffolds. The scaffolds were sputter-coated with gold, and their surface morphology was visualized by SEM at an accelerating voltage of 15 kV (S-3000N, Hitachi, Tokyo, Japan).

### 2.4. Shrinkage Ratio of COL and COL-HANF Scaffolds after Cross-linking Treatment

The diameters of the COL and COL-HANF scaffolds were measured both before and after cross-linking treatment to quantify the degree of shrinkage of the scaffolds during cross-linking. The shrinkage ratio of a scaffold was calculated by the following equation [23]:

Shrinkage ratio (%) = [(D_b_ − D_a_)/D_b_] × 100% (1)

where D_b_ and D_a_ are the diameter of the scaffold before and after cross-linking treatment, respectively.

### 2.5. Compression Test

Compression testing of the scaffolds (approximately 10 mm in diameter, 5 mm in height) was performed at a crosshead speed of 1 mm/min using a material testing machine (ElectroForce^®^ 3100, Bose Cooperation, Eden Prairie, MN, USA). The compressive modulus of the COL and COL-HANF scaffolds was determined from the compression curve for three samples in each group.

### 2.6. Cellular Troliferation on Scaffolds

The COL and COL-HANF scaffolds were UV sterilized overnight and subsequently placed in 24-well tissue culture dishes containing a suspension of MG63 cells (BCRC no. 60279) (1 × 10^5^ cells/well) in MEM supplemented with ascorbic acid (50 µg/mL), ß-glycerophosphate (10 mM), penicillin (100 U/mL), streptomycin (100 μg/mL) and fetal bovine serum (10%) in a humidified atmosphere with 5% CO_2_ at 37 °C. The cultures of cell-seeded scaffolds were harvested on days 1 and 7 to assess the cell proliferation using MTT assays. The cell-seeded scaffolds were incubated with 0.5 mg/mL MTT (3-(4,5-dimethylthiazol-2-yl)-2,5-diphenyltetrazolium bromide) at 37 °C for 4 h. After removal of the supernatants, dimethyl sulfoxide (DMSO) was added, and the quantity of formazan was determined by measuring the absorbance at 570 nm using a microplate-reading spectrophotometer (uQuant, Biotek, Winooski, VT, USA). The cell proliferation rate was calculated according to the following formula:

Proliferation rate = OD_570_ on day 7/OD_570_ on day 1 (2)

### 2.7. ALP Activity of MG63 on the COL and COL-HANF Scaffolds

At a selected timepoint, the cell-containing scaffold was washed several times with phosphate-buffered saline (PBS) and immersed in a cell lysis buffer composed of 0.1 M glycine, 1 mM MgCl_2_ and 1% Triton X-100 in PBS. According to the manufacturer’s protocol for the alkaline phosphatase activity assay (ALP Detection Kit, Randox, Antrim, UK), an appropriate volume ratio of cell lysate and ALP reagent was mixed and incubated at 37 °C for 10 min. The absorbance of the solution was determined at 405 nm. The relative ALP activity, as indicated by the absorbance at 405 nm, was normalized by the absorbance of MTT at 570 nm.

### 2.8. Cytoskeletal Organization and Immunofluorescence

Immunofluorescence staining was used to investigate the cellular morphology inside the scaffolds. The cell-containing scaffolds were collected at various time intervals, fixed with 3.7% paraformaldehyde in PBS for 10 min and subsequently immersed in 0.1% Triton X-100 in PBS for 5 min. Next, the scaffolds were immersed in PBS containing 1% bovine serum albumin for 1 h to avoid nonspecific binding. Afterward, the scaffolds were incubated with primary antibodies against the osteoblast-specific marker protein OCN (AB10911, Millipore, Temecula, CA, USA) or BSP (AB1854, Millipore) to evaluate the level of osteoblast differentiation. Following incubation with the primary antibodies, secondary antibodies (goat anti-rabbit antibody-conjugated rhodamine, sc2091, Santa Cruz, Dallas, TX, USA) were incubated with the scaffolds for 30 min. The immunofluorescence images of the cellular cytoskeleton were examined under a laser scanning confocal microscope (LSCM, LSM 510 META, Zeiss, Oberkochen, Germany). Osteoblast-specific marker proteins and nuclei were stained red and blue, respectively.

### 2.9. Animal Study

The animal experiment was approved by the Institutional Animal Care and Use Committee of National Taiwan Ocean University (approval ID: 104047). Adult male New Zealand rabbits, each approximately 3.0 kg in body weight, were used to evaluate the bone regeneration potential of the COL and COL-HANF scaffolds in femoral condyles. Before implantation, the COL-HANF scaffolds were sterilized by ultraviolet irradiation. The animals were anesthetized using Zoletil 50/Rompun (20 mg/kg and 0.5 mg/kg, intramuscular injection). After shaving and disinfecting the area with iodine, a longitudinal lateral incision was made in the lateral femoral condyle. A critical-sized condyle defect (6 mm diameter) was created using an electric drill (drill diameter = 6 mm) under irrigation with sterile normal saline solution to remove abraded particles during the drilling procedure. The COL-HANF scaffolds were placed into the cavity of the femoral condyle. The incision was closed layer by layer using nylon sutures. Three-dimensional images of the femoral condyle were captured with a microcomputed tomography (µCT) scanner (Skyscan 1174, Bruker, Kontich, Belgium) at 8 weeks after implantation. The bone volume fraction (BV/TV, %), trabecular thickness (Tb.Th, µm) and trabecular separation (Tb.Sp, µm) were calculated using BoneJ plugin for ImageJ software [24].

### 2.10. Statistical Analyses

The results are expressed as the mean ± standard deviation. Statistical analyses were performed using SPSS v.10 software (SPSS Inc., Chicago, IL, USA). A nonparametric Mann-Whitney U test was used to compare the effects of HANFs with those of COL alone. The differences were considered statistically significant at *p* < 0.05.

## 3. Results and Discussion

### 3.1. Characterization of the HANFs

**Figure 1:**
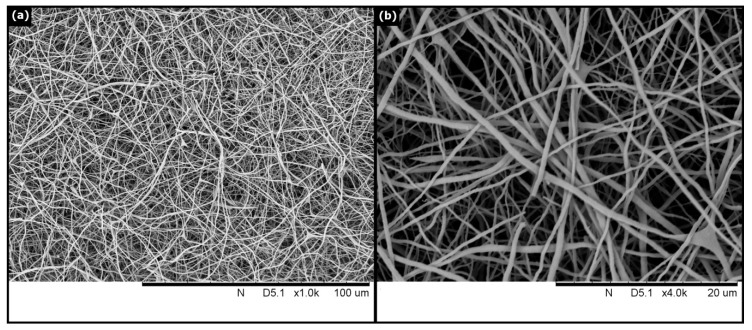
SEM micrograph of the HANFs. (**a**) 1000×. (**b**) 4000×.

**Figure 2:**
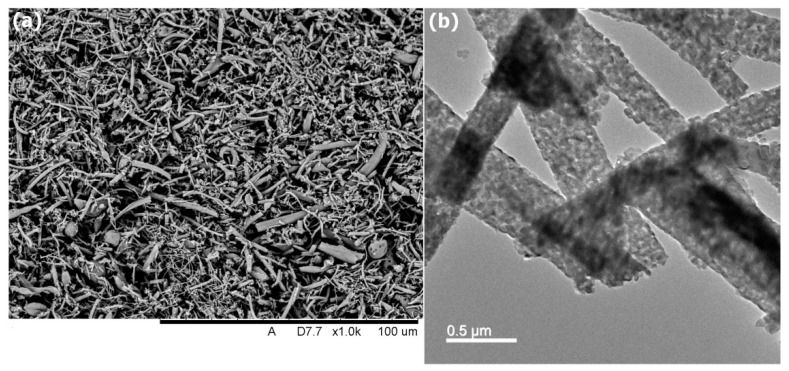
(**a**) SEM and (**b**) TEM images of the HANF fragments.

**Figure 3:**
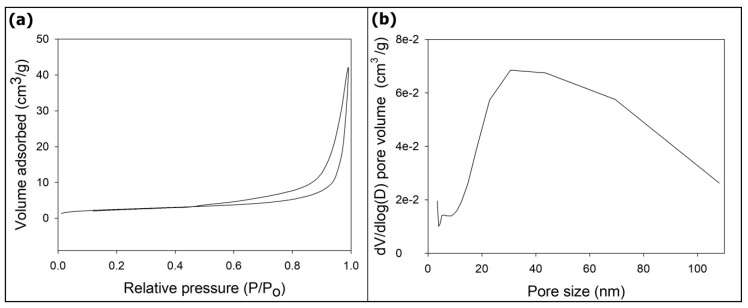
(**a**) N_2_ adsorption-desorption isotherm and (**b**) pore size distribution curve of the HANFs.

The morphology of the HANFs was observed by SEM. Figure 1 shows an SEM image of the HANFs. The diameters of the HANFs ranged between 200 and 800 nm. The fiber diameter is reported as the median (1st quartile, 3rd quartile). The fiber diameter (nm) of the HANFs was 444 (296, 593). The average diameter of the HANFs was approximately 461 ± 186 nm. Figure 2 shows the SEM and TEM images of HANF fragments. From the SEM image (Figure 2a), the lengths of the HANF fragments were approximately 1~50 µm. Moreover, The HANFs were composed of a number of nanocrystals and clearly contain mesopores within the fibers from the TEM image (Figure 2b). The nitrogen gas adsorption-desorption isotherm was shown in Figure 3a. The nitrogen gas adsorption-desorption isotherm of the HANFs was a type IV hysteresis loop which is characteristic of mesoporous material according to the International Union of Pure and Applied Chemistry (IUPAC) classification [25]. The BET specific surface area and total pore volume of HANFs were approximately 8.62 m^2^/g and 0.065 cm^3^/g, respectively. Figure 3b illustrates the pore size distribution curve calculated from the adsorption branch by the Barrett–Joyner–Halenda model. The average pore size was approximately 30 nm.

**Figure 4:**
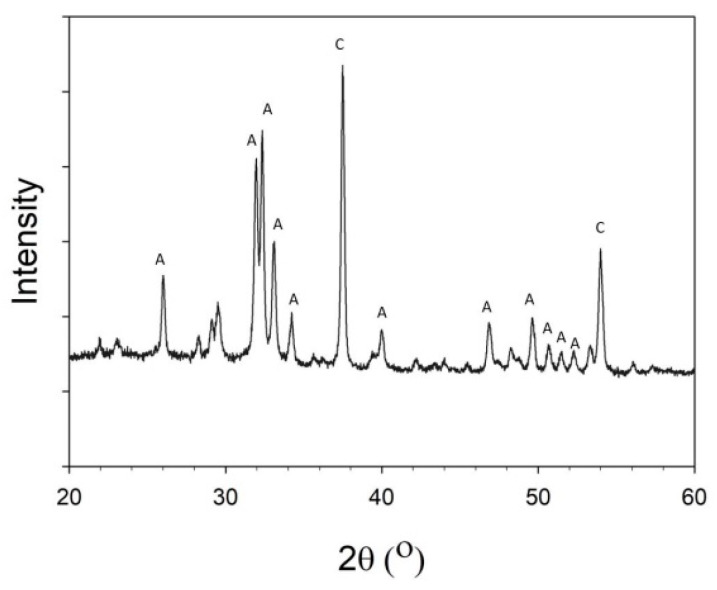
XRD pattern of the HANFs. (C: CaO, PDF 70-4068; A: HAp, PDF 84-1998).

Figure 4 shows a wide-angle XRD patterns of the HANFs. The main crystal phase of the HANFs comprised HAp and CaO. Hatzistavrou [26] found that HAp-CaO composites accelerated the formation of carbonate HAp in simulated body fluids more effectively than pure HAp did. Hence, HAp-CaO composites have been used as bone graft materials with properties that are tunable by varying their composition.

### 3.2. Characterization of the COL-HANF Scaffolds

**Figure 5:**
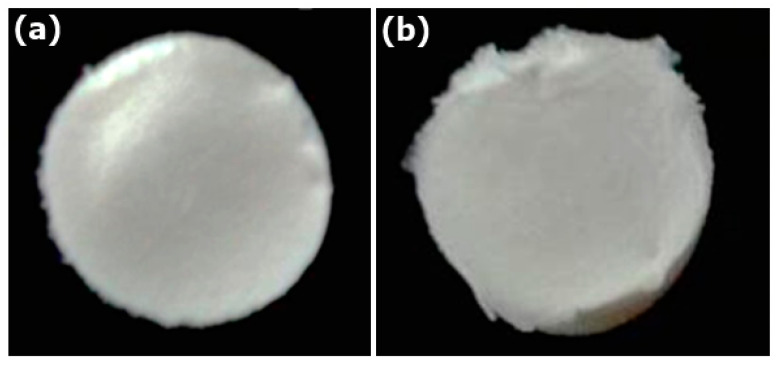
Optical images of the (**a**) COL scaffold and (**b**) COL-HANF scaffold.

**Figure 6:**
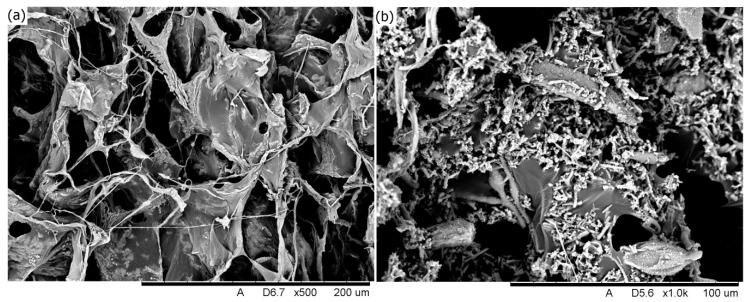
SEM images of the (**a**) COL scaffold and (**b**) COL-HANF scaffold.

**Figure 7:**
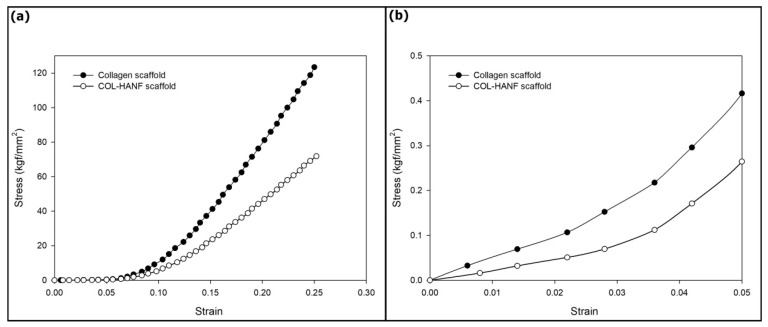
(**a**) Typical compressive stress-strain curves for the COL and COL-HANF scaffolds. (**b**) Expanded view in the low-strain region.

The COL and COL-HANF scaffolds were optically imaged with a mold shape (shown in Figure 5). The microstructures of the COL and COL-HANF scaffolds were highly porous with interconnected pores. Moreover, the HANF fragments were dispersed throughout the COL matrix (as shown in Figure 6). The degree of shrinkage during cross-linking treatment was 24 ± 3.5% and 1 ± 0.1% for the COL and COL-HANF scaffolds, respectively. The HANFs in the COL-HANF scaffold acted as obstacles to prevent shrinkage of the scaffold during cross-linking. [27] The compressive modulus values (Figure 7) of the COL and COL-HANF scaffolds at low strain values after cross-linking treatment were 50.7 ± 11.01 and 22.5 ± 3.79 kPa (n = 3), respectively. At high strain, the compressive modulus values of the COL and COL-HANF scaffolds after cross-linking treatment were 8325.0 ± 163.5 and 4766.8 ± 2.68 kPa (n = 3), respectively. The mechanical properties of the scaffold are related to its pore size, porosity and components. [28,29] Kane et al. noted that COL sponges reinforced with HAp have a higher compressive modulus than unreinforced COL scaffolds. [30] In our past research, we found that reinforcing a COL sponge with mesoporous bioactive glass nanofibers suppressed the shrinkage of the COL scaffold during cross-linking treatment and led to a larger pore size and pore volume fraction than those observed in the COL scaffold. [19] Gleeson et al. [31] showed that the addition of 50 wt % HAp relative to COL resulted in a larger compressive modulus than that observed for the COL scaffold before chemical cross-linking treatment, but the compressive modulus of the HAp-COL scaffold was smaller than that of the COL scaffold after cross-linking treatment. This result is similar to our compressive analysis results.

### 3.3. Cellular Behavior on the Scaffold

**Figure 8:**
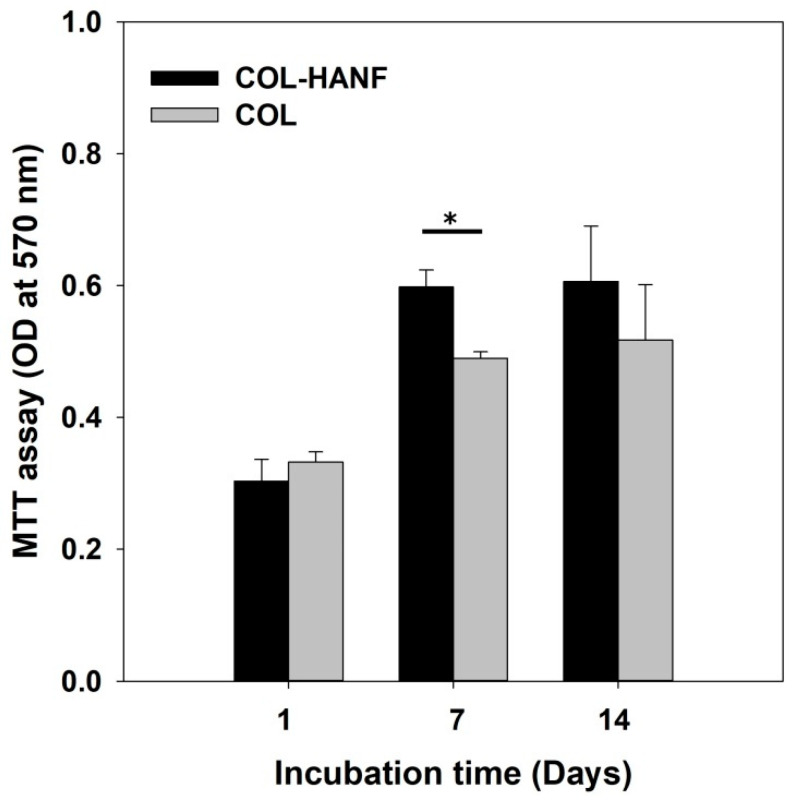
Proliferation of MG63 cells on the COL and COL-HANF scaffolds for up to 14 days of culture. Data are presented as the mean ± SD, n = 3. (*) denotes a significant difference (*p* < 0.05) between the COL scaffold and the COL-HANF scaffold.

Cell proliferation is sensitive to the surface topography and composition of a scaffold [32,33]. The proliferation rates of MG63 cells on the COL and COL-HANF scaffolds were quantified using MTT assays. The MG63 cells incubated on the COL-HANF scaffold showed significantly higher proliferation rates (1.97) than did those incubated on the COL scaffolds (1.47) (as shown in Figure 8). Numerous studies have also found that the incorporation of nano-HAp into scaffolds significantly promotes cell attachment and proliferation [34,35,36].

**Figure 9:**
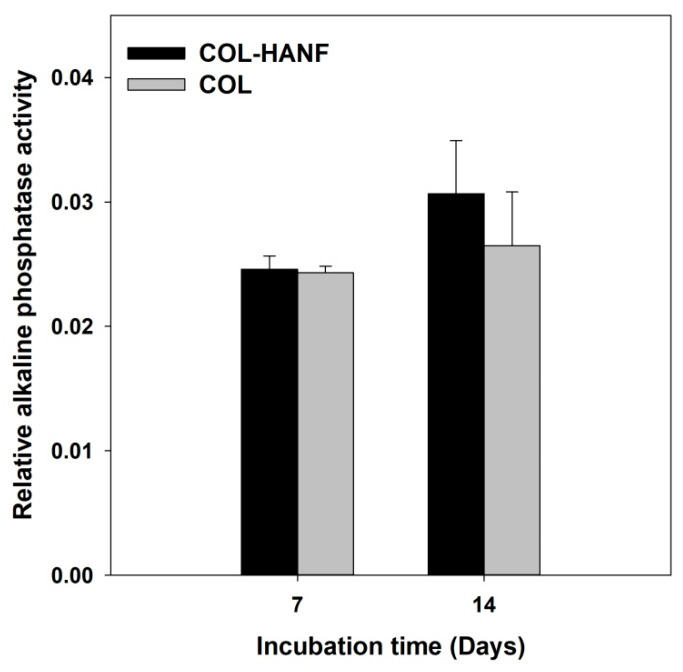
Relative ALP activity of MG63 cells on the COL and COL-HANF scaffolds for up to 14 days of culture. Data are presented as the mean ± SD, n = 3. Statistical analysis was performed to compare the COL and COL-HANF scaffolds. * represents significance at *p* < 0.05 by a nonparametric Mann-Whitney U test.

To evaluate the osteogenic differentiation of MG63 osteoblast-like cells on the scaffolds, the ALP activity and OCN and BSP expression were analyzed. ALP is considered an early marker of osteoblast differentiation. [37] MG63 osteoblast-like cells cultured on the COL-HANF and COL scaffolds were evaluated by assaying the ALP activity after culturing for 14 days. The COL-HANF scaffold showed slightly higher ALP activity than the COL scaffold (shown in Figure 9). Zhou also found that compared with a COL matrix alone, the presence of HAp particles in the COL matrix enhanced the ALP activity of osteoblastic cells. [38] However, in this study, no statistically significant differences were observed between the COL-HANF scaffold and the COL scaffold.

BSP is a noncollagenous phosphorylated glycoprotein in the bone matrix that is involved in the nucleation of HA at the mineralization front of bone [39]. OCN, which is a bone-specific protein synthesized by osteoblasts during the matrix mineralization stage, is thought to bind to HAp and regulate crystal growth through γ-carboxylate groups in an α-helical array [40]. Hence, BSP and OCN are valuable markers of osteogenic differentiation in the middle and mature stages [41].

**Figure 10:**
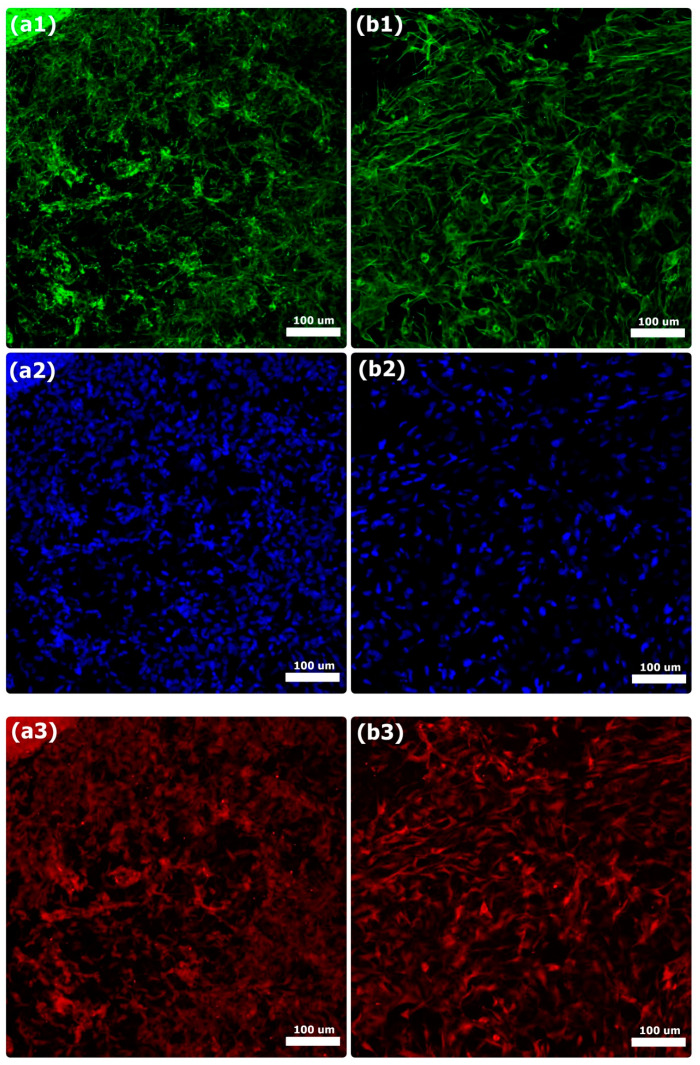
Immunofluorescence analysis of BSP on the (**a1**–**a3**) COL scaffold and (**b1**–**b3**) COL-HANF scaffold after 14 days of culture. MG63 osteoblast-like cells were stained with BSP (red), cell nuclei were stained with DAPI (blue), and cytoskeletal F-actin was stained with FITC (green). Scale bar = 100 μm.

**Figure 11:**
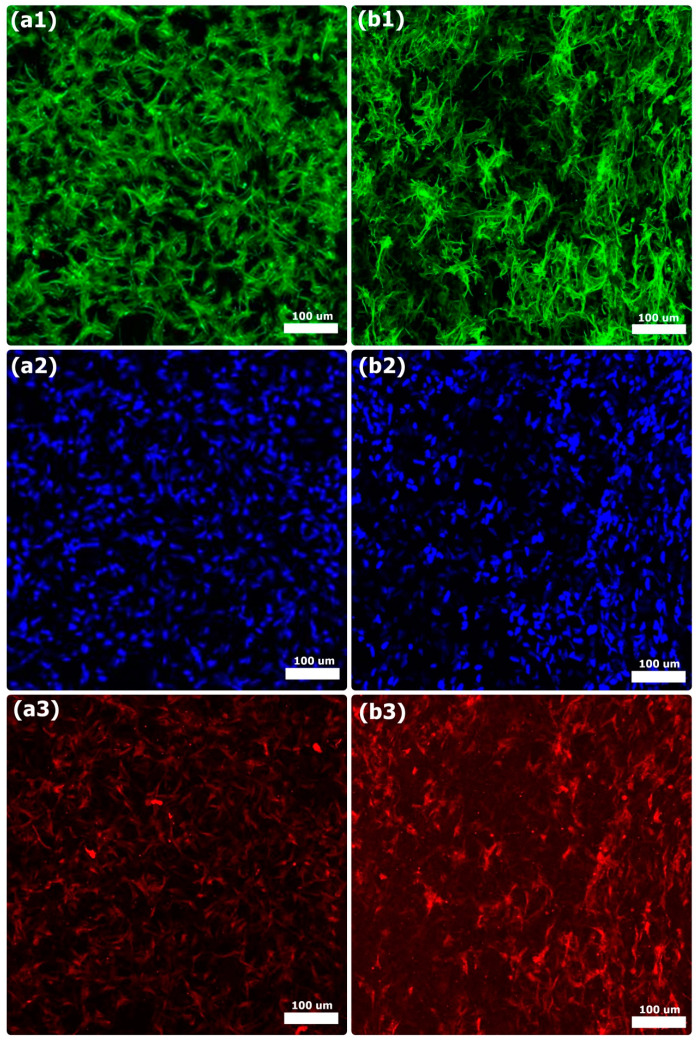
Immunofluorescence analysis of OCN on the (**a1**–**a3**) COL scaffold and (**b1**–**b3**) COL-HANF scaffold after 14 days of culture. MG63 osteoblast-like cells were stained with OCN (red), cell nuclei were stained with DAPI (blue), and cytoskeletal F-actin was stained with FITC (green). Scale bar = 100 μm.

BSP and OCN protein expression on the COL and COL-HANF scaffolds was observed by immunofluorescence staining of MG63 osteoblast-like cells. Both the COL and COL-HANF scaffolds expressed the BSP and OCN proteins on day 14 after incubation. Compared with that in cells cultured on the COL scaffolds, the BSP and OCN protein expression in cells cultured on the COL-HANF scaffolds was higher on day 14 (shown in Figure 10 and Figure 11). Chen et al. found that osteoblasts cultured on chitosan scaffolds containing nanohydroxyapatite exhibited better osteogenic marker production than those cultured on chitosan-only scaffolds [42]. Ikeda et al. noted that the osteogenic differentiation of SaOS-2 osteoblastic cells was accelerated on HAp/COL compared to COL [43]. Jung et al. showed that calcium ion release from HAp improved MC3T3-E1 cell differentiation by increasing the expression of BSP and OPN through L-type calcium channels, which triggered the CaM–CaMK2 pathway [44]. These results indicate that the presence of HANFs in the COL-HANF scaffold increased the osteogenic marker production compared with that on the COL scaffold.

### 3.4. Microcomputed Tomography (Micro-CT) Evaluation

**Figure 12:**
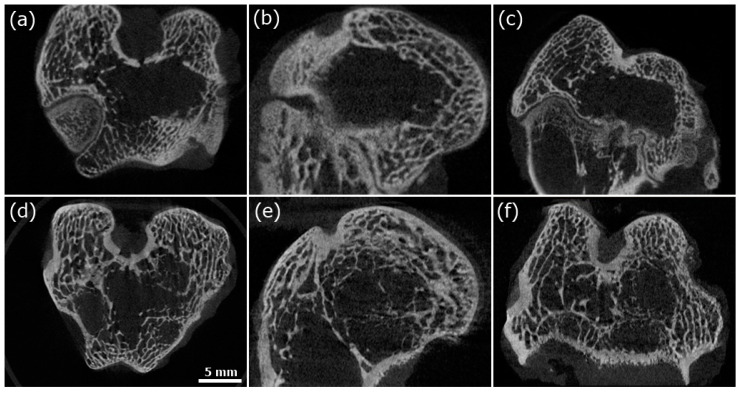
Micro-CT sections of the unimplanted group (**a**–**c**) and COL-HANF group (**d**–**f**). (**a**,**d**) Horizontal plane. (**b**,**e**) Sagittal plane. (**c**,**f**) Coronal plane.

To evaluate new bone formation in femoral condylar bone defects of New Zealand white rabbits, a micro-CT scan was performed 2 months after the operation (shown in Figure 12). The BV/TV, Tb.Th and Tb.Sp values were 16.59 ± 5.11%, 0.230 ± 0.005 mm, and 0.548 ± 0.212 mm, respectively, in the unimplanted group and 19.97 ± 3.50%, 0.260 ± 0.019 mm, and 0.710 ± 0.173 mm, respectively, in the COL-HANF scaffold group (shown in Table 1). In addition, more new bone formation in the center of the bone defect could be observed in the COL-HANF scaffold group than in the unimplanted group. These results demonstrated that the COL-HANF scaffold possessed better bone regeneration activity than that observed in the unimplanted group. Eight weeks after implantation, the condylar bone defect was not completely regenerated, and the COL-HANF scaffold had been completely absorbed. From our past research, HANFs have a high drug-loading efficiency due to their mesoporous structure [20]. Wang et al. demonstrated that COL-HAp with bone morphogenetic proteins (BMPs) promoted faster and more extensive new bone formation than COL-HAp [45]. Hence, the addition of growth factors (such as BMPs) to the COL-HANF scaffold will accelerate the regeneration of bone defects to overcome the mismatch between the degradation of the COL-HANF scaffold and the recovery of bone defects.

## 4. Conclusions

A HAp-CaO fiber fragments-COL composite scaffold was developed for use in bone grafts. HANFs decreased the degree of matrix shrinkage from 24 ± 3.5% for the pure COL scaffold to 1 ± 0.1% for the composite scaffold. However, at low or high strain, the compression moduli of COL-HANF were lower than those of COL because the content of HANF relative to COL was more than 50 wt %, resulting in a larger pore size and pore volume. Cells incubated with the COL-HANF scaffold exhibited higher ALP activity and expressed higher levels of BSP and OCN on day 14 than did cells incubated with the COL scaffold. Together, the cell proliferation and differentiation results indicated that the COL-HANF scaffold can promote osteoblast cell differentiation until the final mineralization stage. In an animal study, the regeneration of bone defects after COL-HANF scaffold implantation was better than that in the control group (unimplanted) at eight weeks after surgery. Clearly, the in vitro and in vivo properties examined herein show the potential of COL-HANF scaffolds in bone tissue regeneration.

## Figures and Tables

**Table 1:** Bone volume/total volume (BV/TV), trabecular thickness (Tb.Th) and trabecular separation (Tb.Sp) for defects implanted with the COL-HANF scaffold and for unfilled defects (control group). Mean ± SD; n = 3.

	BV/TV (%)	Tb.Th (mm)	Tb.Sp (mm)
COL-HANF	19.97 ± 3.50	0.260 ± 0.019 mm	0.710 ± 0.173
Control group	16.59 ± 5.11	0.230 ± 0.005	0.548 ± 0.212
